# Detecting Network Communities: An Application to Phylogenetic Analysis

**DOI:** 10.1371/journal.pcbi.1001131

**Published:** 2011-05-05

**Authors:** Roberto F. S. Andrade, Ivan C. Rocha-Neto, Leonardo B. L. Santos, Charles N. de Santana, Marcelo V. C. Diniz, Thierry Petit Lobão, Aristóteles Goés-Neto, Suani T. R. Pinho, Charbel N. El-Hani

**Affiliations:** 1 Institute of Physics, Federal University of Bahia, Campus Universitário de Ondina, Salvador, Bahia, Brazil; 2 Institute of Mathematics, Federal University of Bahia, Campus Universitário de Ondina, Salvador, Bahia, Brazil; 3 National Institute for Space Research, São José dos Campos, São Paulo, Brazil; 4 Mediterranean Institute of Advanced Studies, IMEDEA (CSIC-UIB), Esporles (Islas Baleares), Spain; 5 Department of Biological Sciences, State University of Feira de Santana, Feira de Santana, Bahia, Brazil; 6 Institute of Biology, Federal University of Bahia, Campus Universitário de Ondina, Salvador, Bahia, Brazil; King's College London, United Kingdom

## Abstract

This paper proposes a new method to identify communities in generally weighted complex networks and apply it to phylogenetic analysis. In this case, weights correspond to the similarity indexes among protein sequences, which can be used for network construction so that the network structure can be analyzed to recover phylogenetically useful information from its properties. The analyses discussed here are mainly based on the modular character of protein similarity networks, explored through the Newman-Girvan algorithm, with the help of the neighborhood matrix 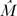. The most relevant networks are found when the network topology changes abruptly revealing distinct modules related to the sets of organisms to which the proteins belong. Sound biological information can be retrieved by the computational routines used in the network approach, without using biological assumptions other than those incorporated by BLAST. Usually, all the main bacterial phyla and, in some cases, also some bacterial classes corresponded totally (100%) or to a great extent (>70%) to the modules. We checked for internal consistency in the obtained results, and we scored close to 84% of matches for community pertinence when comparisons between the results were performed. To illustrate how to use the network-based method, we employed data for enzymes involved in the chitin metabolic pathway that are present in more than 100 organisms from an original data set containing 1,695 organisms, downloaded from GenBank on May 19, 2007. A preliminary comparison between the outcomes of the network-based method and the results of methods based on Bayesian, distance, likelihood, and parsimony criteria suggests that the former is as reliable as these commonly used methods. We conclude that the network-based method can be used as a powerful tool for retrieving modularity information from weighted networks, which is useful for phylogenetic analysis.

## Author Summary

Complex weighted networks have been applied to uncover organizing principles of complex biological, technological, and social systems. We propose herein a new method to identify communities in such structures and apply it to phylogenetic analysis. Recent studies using this theory in genomics and proteomics contributed to the understanding of the structure and dynamics of cellular complex interaction webs. Three main distinct molecular networks have been investigated based on transcriptional and metabolic activity, and on protein interaction. Here we consider the evolutionary relationship between proteins throughout phylogeny, employing the complex network approach to perform a comparative study of the enzymes related to the chitin metabolic pathway. We show how the similarity index of protein sequences can be used for network construction, and how the underlying structure is analyzed by the computational routines of our method to recover useful and sound information for phylogenetic studies. By focusing on the modular character of protein similarity networks, we were successful in matching the identified networks modules to main bacterial phyla, and even some bacterial classes. The network-based method reported here can be used as a new powerful tool for identifying communities in complex networks, retrieving useful information for phylogenetic studies.

## Introduction

In networks, module or community structure plays a central role when it comes to understand network topology and dynamics. To advance solutions to many problems related to biological networks, we need to identify, thus, the community structure in datasets. Consequently, the introduction of new efficient and robust methods that are able to perform such a task in a variety of situations is of utmost importance.

We are interested, here, in giving a contribution to the complex issue of phylogenetic inference by appealing to the complex network approach, which has been successfully applied to uncover organizing principles that govern the constitution and evolution of various complex biological, technological, and social systems [1]–[4]. Recent studies using complex network approaches in the fields of both genomics and proteomics have contributed to a better knowledge of the structure and dynamics of the complex webs of interactions of a living cell [5]–[12]. Several kinds of biologically relevant networks have been studied in the last years, mainly protein interaction, transcriptional, and metabolic networks [1]. In this study, we work with another set of relationships, namely, the evolutionary relationships between proteins throughout phylogeny, and introduce a new method to identify communities in generally weighted complex networks.

The reliability and overall applicability of a new proposed method is the subject of a long term research program, which necessarily starts with a clear formulation of the key steps of the method, alongside with the analysis of a non trivial problem that has been analyzed before, such as, for instance, phylogenetic inference.

There are four families of methods of phylogenetic analysis that are commonly used, namely: maximum parsimony, distance, maximum likelihood, and Bayesian [13]. Promising prospects of developing new trustful methods to infer phylogenetic relationships are offered by the possibility of using primary information about protein sequences contained in open access databases and the derived protein similarity measures. We introduce here a methodology to identify community structure in such primary data sets, based on the concept of distance between complex networks, and apply it to the specific problem of retrieving useful information that can be used to infer phylogenetic relationships. In this process, we avoid as much as possible the use of any qualitative pre-existing biological information. We show here that a method based on complex network theory can recover information about the evolutionary relationships between organisms, as expressed in the similarities and differences between their protein or DNA sequences.

Depending on the way the nodes are connected within a network, it may be possible to identify one or more subsets of nodes such that the average number of connections among nodes within any of these subsets is distinctly larger than the average number of connections with nodes outside this subset. The identification of such subsets (usually called communities, modules, components, clusters, etc.), a key issue that has not been completely solved within complex network theory, is of utmost importance for biological applications. Indeed, modular properties are found to be very common features in any branch or level of biological network investigations.

Over the past years, the amount of research in identifying communities in networks is really astonishing. There are several review articles discussing this subject, based on mathematical and computational approaches [14]–[16]. Furthermore, comparative analyses of the available methods are also found in the literature [17], [18].

Computationally efficient approaches based on similarity matrices and cluster analyses for the exploration of protein databases with little or no prior knowledge are important tools for phylogenetic analysis. A number of approaches are currently being used to infer evolutionary relationships between proteins. For instance, the Markov Cluster (MCL) Algorithm [19], [20] is an unsupervised cluster algorithm that has been applied to the analysis of graphs in several different domains, mostly in bioinformatics. The MCL Algorithm was used, for instance, for the detection of protein families [21], a major research goal in structural and functional genomics. MCL was also extended to the identification of orthologous groups by OrthoMCL [22]. It was also used to develop phylogenomic analyses of specific taxa, such as the Ascomycota [23]. A hybrid approach to sequence-based clustering of proteins was developed, combining Markov with single-linkage clustering, with the intention of obtaining both specificity (as allowed by MCL) and the preservation of topological information as a function of threshold information about protein families (as in single-linkage clustering) [24]. Another recently developed method for automatic and unsupervised detection of protein families and genome annotation is the Global Super Paramagnetic Clustering (SPC) Algorithm, which showed higher accuracy, specificity and sensitivity of clustering than MCL [25]. Finally, Kóvacs et al. [18] introduced ModuLand, an integrative network module determination method family, which can determine overlapping network modules as hills of an influence function-based, centrality-type community landscape. The new method to identify communities in generally weighted complex networks proposed here is quite powerful and innovative in the use of a distance *δ* (to be defined in the next section) to determine an optimal value of the threshold on similarity.

Two main tasks are crucial to derive an objective, mathematically based community identification: First, to define a measure suitable to distinguish non-modular from modular character, and, second, to identify the communities, when this is the case. The distance *δ* used herein is able to help the identification of the modular character in a very clear way. Therefore, our major contribution, based on complex network theory, is to use this measure together with the protein similarity matrix (in fact, the weight matrix of any weighted network) to identify the minimal set of links that are included in the network in order to preserve the relevant biological information necessary to unveil the modular character within the data set at stake.

Once such optimally chosen network is found, any proposed community detection method may be used to retrieve the existing communities. We use here the Newman Girvan algorithm (NGA) [26], which, although time consuming, also allows to identify the sequence of branching events, leading to useful and well defined dendrograms.

Since several organic biomolecules are required for basic metabolic purposes, they can be found in large number of organisms, making it possible to use techniques derived from complex network theory to explore information that is useful for phylogenetic inferences. Enzymes that are involved in the synthesis of ubiquitous and metabolically important molecules seem particularly promising for such complex network approach. They are likely to be found in many distinct organisms and, if they are involved in ancient metabolic pathways, they can be found in the three life domains, Archaea, Bacteria, and Eukarya. Even though distinct organisms use their own enzyme variants to produce a given molecule, these variants will tend to look more similar in their amino acid sequences the closer the species are in phylogenetic terms. Thus, species can be gathered in phylogenetically meaningful groups by analyzing the degree of similarity of enzymes involved in some basic metabolic pathway. We show here how the similarity of the amino acid sequences of enzymes derived from completely sequenced genomes of extant organisms can be used for network construction and, subsequently, the network structure can be analyzed so as to recover phylogenetically useful information from its properties and statistics.

The methods described here can be used for any set of proteins involved in basic metabolic pathways. We will work in this paper with data from enzymes involved in chitin synthesis. Chitin, the β-1,4-linked linear homopolymer of N-acetylglucosamine, is a structural endogenous carbohydrate, which is a major component of fungal cell walls [27], cephalopod beaks [28], integuments of larvae and young nematodes [29], and arthropod exoskeletons [30]. Chitin is the second most abundant polysaccharide in nature after cellulose. It occurs only in eukaryotic organisms of the Metazoa-Fungal clade. This suggests that chitin may have evolved before the crown eukaryotic radiation.

Chitin is synthesized by a sequence of six successive reactions: (i) conversion of Glu-6P into Fru-6-P by phosphoglucoisomerases (E.C. 5.3.1.9); (ii) conversion of Fru-6-P into GlcN-6-P by glucosaminephosphate isomerases (E.C. 2.6.1.16); (iii) acetylation of GlcN-6-P generating GlcNAc-6-P by phosphoglucosamine acetylases (E.C. 2.3.1.4), (iv) interconversion of GlcNAc-6-P into GlcNAc-1-P by acetylglucosamine phosphomutases (E.C. 5.4.2.3) or, alternatively, by acetylglucosamine phosphate deacetylases (E.C. 3.5.1.25); (v) uridilation of GlcNAc-1-P by UDP-acetylglucosamine pyrophosphorylases (E.C. 2.7.7.23); and (vi) conversion of UDP-GlcNAc into chitin by chitin synthases (E.C. 2.1.4.16) [31], [32].

Chitin degradation is achieved by chitinases (E.C. 3.2.1.14), either by exochitinases, which convert chitin into N-acetylglucosamine residues, or by endochitinases, which convert chitin into chitobiose, which, in turn, may be converted into N-acetylglucosamine residues by hexoaminidases (E.C. 3.2.1.52). N-acetylglucosamine residues may be activated by acetylglucosamine kinases (E.C. 2.7.1.59) to form N-acetylglucosamine-6-P, restoring the precursor of the short feedback cycle of chitin metabolism. Chitin may also be deacetylated by chitin deacetylases (E.C. 3.5.1.41), converted into chitosan, which is degraded by chitosanases (E.C. 3.2.1.132) into glucosaminide, which, when converted into glucosamine, may be activated by hexokinase type IV glucokinases (E.C. 2.7.7.1), which restore the precursor of N-acetylglucosamine-6-P, Glucosamine-6-P, configuring a longer feedback cycle [33].

Even though chitin itself is found only in the Metazoa-Fungal clade, we can find proteins which are homologous to enzymes involved in chitin synthesis in other clades, including bacterial and archaeobacterial ones. Therefore, the chitin metabolic pathway can be used to recover phylogenetically relevant information in the three life domains.

In this paper, we use the complex network approach as a theoretical and methodological tool to perform a comparative study of the enzymes related to the chitin metabolic pathway in extant organisms of the three life domains, Archaea, Bacteria, and Eukarya. We will show how the information derived from the network structure and statistics can be used to uncover phylogenetically useful modules, retrieving sound biological information by computational routines, without using biological assumptions other than those incorporated by BLAST.

## Methods

### Database and comparative analysis

Our primary database consists of protein sequences of completely sequenced genomes of extant organisms that can be freely accessed at the GenBank - NCBI [34] (http://www.ncbi.nlm.nih.gov/Genbank/). Protein data provide essential information to the identification of any given organism, as well as to comparative studies on evolutionary paths followed by different organisms. Our data set, downloaded from GenBank at May 19th, 2007, contains information from 1695 organisms. We used completely sequenced genomes to assure that all putative proteins and their isoforms, if any, could be adequately retrieved [35].

We developed automatic procedures to filter the protein related data in the complete downloaded database. In the first step of the process, we extracted from the primary database the relevant information for the current work, namely, the molecular source of protein sequences, their structural and functional information, and the taxonomic classification of the organisms in which the proteins are found. Next, we scrutinized the secondary database obtained in this manner, in order to identify which proteins (i.e., the organism-specific protein variants that play the same biological function) are present in a large number of organisms. One way to optimize this search, in the sense of finding many organisms with the same protein, is to pre-select a basic biomolecule, such as chitin, and look for the enzymes involved in its metabolism. Indeed, our search revealed that some of the proteins with the largest number of entries in the database are enzymes that take part in the metabolic synthesis or degradation of chitin. In Table 1, we indicate five such enzymes, satisfying the condition of being present in more than 100 organisms from the 1695 original set [33]. The remarkably large number of bacterial records in the database reflects the fact that there are much more completely sequenced organisms of the Bacteria domain than of the Archaea and Eukarya domains.

**Table 1:** Enzymes associated with the chitin metabolic pathway that satisfy the condition of being present in more than 100 organisms from the 1695 original data set, downloaded from GeneBank at May 19^th^, 2007.

Protein	E.C. number	Domain (#)
Acetylglucosamine phosphate deacetylase	3.5.1.25	B(170), A(6)
Glucosaminephosphate isomerase	2.6.1.16	E(23), B(285), A(5)
Hexosaminidase	3.2.1.52	E(3), B(235)
Phosphoglucoisomerase	5.3.1.9	E(16), B(472), A(12)
UDP-acetylglucosamine pyrophosphorylase	2.7.7.23	E(2), B(324), A(2)

Abbreviations: E = Eukarya; B = Bacteria; A = Archaea; E. C. = Enzyme commission. Number in parentheses after the letters shows the total of organismic individual sequences per domain for each protein.

After identifying the sets of organisms that possessed each of the proteins listed in Table 1, we used BLAST 2.2.15 [36], with a pairwise alignment, to perform quantitative comparisons among the protein sequences pertaining to each set. From the BLAST outputs, we used in our study the similarity index.

Then, a similarity matrix *S* was constructed based on the similarity level between protein sequences, where any element of the similarity matrix *S_ij_*∈0,100] is the similarity index associated with the protein sequences *i* and *j*. Since *S* is not necessarily symmetric (*S_ij_≠S_ji_*), it is important to consider a symmetric version *S*, where the elements are defined by *S_ij_ = min(S_ij_,S_ji_)*.

The programs were executed both on LINUX- and WINDOWS-running computers. Databases were managed through MySQL. Scripts and auxiliary programs were written in PERL, BASH, C, C++ and FORTRAN 77. PAJEK [37] was used to generate network images.

In the sub-section **Network construction**, we describe how we used ***S*** to generate complex networks depending on a similarity threshold for each one of the five proteins shown in Table 1. Networks were analyzed by the methods described in the sub-section **Network analysis**, while the modular patterns generated by complex network approach were biologically interpreted in the light of the phylogenetic relationships of organisms.

### Network construction

Before defining the networks used in this study, let us recall that the most used characterization of network properties is based on a series of measures [38], including: the number of nodes, *N*; the shortest path *d(i,j)* between nodes *i* and *j*; the average minimal distance 〈*d*〉 taken over all pairs of nodes; the network diameter *D*, defined by the largest value of *d(i,j)*; the node clustering coefficient *c_i_*, which measures how strongly connected the neighbors of node *i* are; the network clustering coefficient *C*, corresponding to the average value over the *c_i_*; the node degree, *k_i_*, defined by the number of links of a node *i* and its average value over all nodes 〈*k*〉; the functional relationships *p(k)*, the probability distribution of nodes with *k* links, and *C(k)*, the distribution of node clustering coefficients with respect to the node degree *k*.

In general, the key step in the construction of a system interaction network is to define a meaningful criterion to place an edge between two nodes, which should be able to identify the presence and strength of the interaction between them. In the current study, the concept of interaction corresponds to protein similarity, which is related, in turn, to the evolutionary relationships between the organisms possessing the proteins at stake [35]. Therefore, the similarity matrix *S* constitutes the starting point to obtain the protein similarity networks (PSN).

In a PSN, the nodes correspond to the protein sequences, and the presence of edges between two nodes depends on how similar the related proteins are. Each network can be defined by its adjacency matrix (AM) *M*, for which any matrix element *m_i,j_* is set to 1, if the nodes *i* and *j* are connected, or to 0, if not. Note that it is straightforward to switch from the AM network description to the list description, in which the network is characterized by a list of L pairs of nodes connected by a link. To be more precise, let us define a network family depending on a threshold value *σ*, where the elements of its adjacency matrix *M(σ)* satisfy:
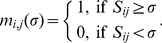
(1)


This strategy makes it possible to replace one single weighted network defined in terms of *S* by a family of unweighted networks, which can be analyzed by a large number of recently developed methods and measures [38]–[41].

Depending on the value of *σ*, the interaction network may be completely distinct: for small values of *σ* it is highly connected, while for large values of *σ* it is poorly connected. As we will show in the next section, we have performed a detailed investigation of the dependence of the network properties on the value of *σ*. We are able to establish a well defined criterion for optimal choices of *σ*, in the sense that the networks generated within a relatively narrow range of values of *σ* display a modular pattern that can be interpreted in phylogenetic terms, as addressed in the section of results and discussion of the present paper.

To fine tune the value of *σ* that makes it possible to unveil the modular character, we use the concept of higher order neighborhoods of a node [42]. Two nodes *i* and *j* are neighbors of order *ℓ* when the shortest path between them consists of *ℓ* edges. In this manner, it is possible to define a *ℓ*-th order neighborhood of a given network represented by *M* if we connect all pairs of nodes that are *ℓ* steps apart. Such networks can be defined in terms of *M(ℓ)*, the corresponding AM of order *ℓ*. The elements of this matrix are defined as:
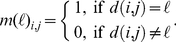
(2)


The knowledge of the set *{M(ℓ)}*, where *ℓ∈[1,D]*, allows us to define the following neighborhood matrix
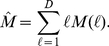
(3)


The matrix elements of 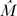, denoted as 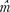 *_i,j_*, indicate the shortest path between the nodes *i* and *j*. If the network is assembled by two or more disjoint clusters, the distance *d(i,j)* between two nodes, say *i* and *j*, belonging to two distinct clusters is ill-defined. In order to sidestep this indeterminacy and continues operating with 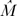, we set 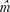 *_i,j_* = 0 whenever this occurs. The importance of 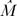 for a deeper analysis of the neighborhood structure of a network has been indicated in a series of previous studies [43]–[45]. The utility of 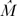 ranges from providing an insightful visualization of the neighborhood structure by means of color plots to defining a distance between pairs of networks [45]. This last measure can be used to identify how similar two networks are. For this purpose we define the distance *δ(α,β)* between any two networks with the same number of nodes (*α* and *β*) by:
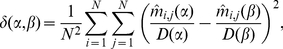
(4)where *D(α)* represents the diameter of the network *α*.

In a general comparison process, the obtained value of *δ(α,β)* depends on the adopted node enumeration for both networks, although the network topology does not depend on it. Therefore, for the purpose of providing a useful measure, the definition (4) can be made more precise by restricting the value of *δ(α,β)* to the minimal value assumed when all possible node enumerations are taken into account (see [45]). In the current study, *α* and *β* are two distinct protein networks, generated by one same dataset, but where the edges are inserted according to Eq. (1) when we consider *α = σ_1_ = σ* and *β = σ_2_ = σ+Δσ*. In this definition, we suppose that *σ_1_* and *σ_2_* are two nearby values of *σ*. Since the nodes represent the same proteins, it is not necessary to consider different enumerations, but just to use the same enumeration to generate both networks. If we plot *δ*(*σ,σ+*Δ*σ*) as function of *σ*, it turns out that the graph is characterized by the presence of sharp peaks. Such series of consecutive values of *δ*(*σ,σ+*Δ*σ*) marks the points where the obtained networks are about to suffer important topological changes [43], i.e., to be split into separate communities.

The value of *σ* plays a key role in the network definition, which is similar to the probability *p* to establish an edge in a random Erdös-Rényi network. By varying the value of *p*, the network changes to an assembly of disconnected edges at *p = 0* to a complete graph when *p = *1. The most interesting situation, however, occurs in the neighborhood of one critical value *p_cr_*≈1*/N*, which is related to the emergence of a giant cluster that contains the overwhelming majority of nodes.

### Network analysis

The investigation reported in this paper is based on the measures defined in the previous subsection, and also in other measures that allow for the identification of modularity properties of the network, if any. Loosely speaking, a module in a network is composed of a sub-set of nodes that are overwhelmingly more connected among themselves than with other network nodes.

The link betweeness degree *b_ij_* between nodes *i* and *j* is the basic concept within the NGA to identify network communities. *b_ij_* counts the fraction of all shortest paths connecting the *N(N−1)/2* pairs of nodes that pass through the *(i,j)* link, providing a quantitative measure of the relevance of each link for the optimized network information traffic. NGA proceeds by sequentially eliminating the edges with largest value of *b_ij_* [26]. As a result, it is possible to obtain a network dendrogram where the number of branches increases with the number *r* of eliminated links. In this way, the dendrogram has one single branch when *r = 0* – in the case of a connected network – and *N* single-node communities when *r = L*. Each value of *r* informs the set of nodes that are still connected in a given cluster. Since this is a time consuming program, faster tracks have been proposed to analyze very large networks [38]–[41], [46]. In the current case, however, we are able to work with this method, given that our networks are not too large.

In our analyses, we used the NGA to identify existing communities for any value of *σ*. As the detected communities may be quite distinct from one value of *σ* to another, the NGA results corroborate our claim that the identification of the optimal value of *σ* using the distance *δ* is the crucial step of the whole procedure.

To reveal the modular structure of the network, NGA requires a node re-enumeration, a step that is also included in our procedure. Therefore, it is possible to use the re-enumerated form of 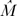 to visualize the modularity of the protein similarity networks with color plots. The modularity structure becomes quite clear when we draw color plots for the elements of 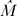 using the same node labeling obtained at the final step of the dendrogram evaluation.

We want to comment further that the concept of distance *δ(α,β)* can also be used to follow the process of link elimination within NGA. In this particular case, *α* and *β* identify two networks characterized by having *m* and *m+1* eliminated links within NGA (see [26]). A graph of *δ(m,m+1)* as a function of *m* indicates, by high peaks, those events of link eliminations that correspond to branching points in the dendrogram. As it was shown in [45], the distance *δ(m,m+1)* is able to indicate the branching points in a much clearer way when compared to, e.g., the modularity function *Q* introduced by Newman and Girvan [26].

As shown in Table 1, we constructed networks for five enzymes of the chitin metabolic pathway, which provided, in turn, different classifications for the organisms included in the database. In order to quantitatively assess the possible differences between the classification provided by the networks based on different enzymes, say *ϕ* and *ψ*, we evaluated a congruence index *G*(*ϕ*,*ψ*) according to the following prescription: i) we count the number *R(ϕ,ψ)* of common organisms that are present simultaneously in both networks; ii) we look for the correspondence between the different communities from *ϕ* and *ψ* that maximizes the number of matching organisms *Q*(*ϕ*,*ψ*), i.e., organisms that are placed in the same communities in the two networks. In doing this, we must observe that, if the number of communities in *ϕ* and *ψ* are different, it is necessary to make a correspondence of two or more communities of network *ϕ* to the same community in network*ψ*. The value *G*(*ϕ*,*ψ*) is defined as the ratio *Q*(*ϕ*,*ψ*)/*R*(*ϕ*,*ψ*).

To conclude, the methodology that is applied to generate the results presented in the next section can be summarized in terms of the following steps:

- Select the protein sequences with the relevant information to set up the similarity level between the sequences.
- Compare the protein sequences using BLAST and set up the *n×n* similarity matrix, being *n* the number of protein sequences.
- Generate a set of networks associated with the chosen values of the similarity threshold (*σ*): the nodes correspond to the protein sequences and a link is inserted between a pair of nodes if the similarity between the proteins is larger or equal to *σ*. In the current case we considered all integer values of *σ* in the interval [0,100].
- Set up the neighborhood matrix 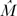 associated with each adjacency matrix.
- Calculate the distance between the networks *δ*(*σ,σ+*Δ*σ*), and select for analysis the critical networks, for which the *δ*(*σ,σ+*Δ*σ*) assumed the local maximal value.
- For the critical networks, apply the Newman Girvan algorithm (NGA), removing the edges with the maximal value of edge betweenness until there is no link at all.
- In order to detect the modular structure of the network, set up the dendrogram for the critical network as well as the color representation of the neighborhood matrix.
- Calculate the congruence index *G*(*ϕ*,*ψ*) to quantitatively assess the differences between the classification provided by the distinct networks.

## Results/Discussion

Here, we present and discuss results concerning the modular structure of protein similarity networks provided by our method that are useful for phylogenetic inferences. To be concise, we provide a detailed discussion of the results obtained for two proteins in Table 1: UDP-acetylglucosamine pyrophosphorylase (to which we will refer below as UDP) and acetylglucosamine phosphate deacetylase (Acetyl). Then, we will provide a comparative analysis of the results for the networks of all the five proteins investigated in this study, in order to provide evidence for the classification consistency of the method.

### Community detection

**Figure 1:**
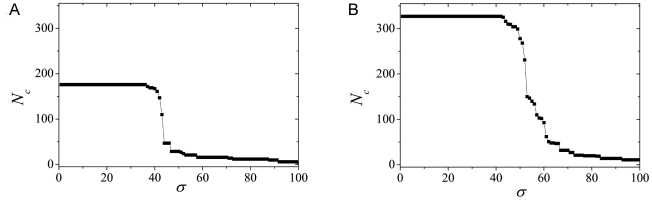
The size of the largest connected component (*N_c_*) versus the threshold similarity *σ*: a) Acetyl; b) UDP.

Let us now illustrate how the behavior of *δ*(*σ,σ+*Δ*σ*) provides a precise way of characterizing the dependence of the networks on *σ* (step (E) in the summary of the methodology presented in the previous section). This behavior is illustrated in Figure 1a for the Acetyl network. The results were obtained by making the values of *σ* differ in *Δσ* = 1%. The graph shows three well defined maxima of *δ*(*σ,σ+*Δ*σ*) for *σ* in the interval [30%,50%], the largest of which occur at *σ = σ_max_* = 42%. These results should be interpreted as follows: if *σ = 0*, the network consists of a completely connected single cluster. By increasing the value of *σ*, we restrict the number of bonds in the network, so that 〈*d*〉 increases together with the values of the matrix elements 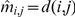. Since the distance *δ*(*σ,σ+*Δ*σ*) makes a comparison of the influence of changing *σ* on *d(i,j)*, a sharp increase in its value indicates that the bond removal is leading to large changes in the values of some of *d(i,j)*. This suggests also that important network topological changes are about to occur. The most drastic events, expressed by the first sharp peaks, are usually related to the disassembling of one large set of nodes (module) from the original, completely connected cluster. This network, which we will call the critical network, is selected to be analyzed. Later on, smaller peaks indicate the splitting of larger modules into smaller ones. This occurs when the last bonds linking these modules to the network are removed. The very high peak at *σ = σ_max_* = 42% indicates that a large topological change occurred at this particular value.

The same scenario is observed in Figure 1b for the *δ*(*σ,σ+*Δ*σ*) results obtained from the UDP network. Note that the peaks occur at higher values of *σ*, in comparison to the Acetyl network, and a richer structure of peaks of comparable sizes is found. Despite these quantitative changes, the two graphs show similar features, representing the kinds of structural changes in the network due to the variation of the threshold similarity value.

**Figure 2:**
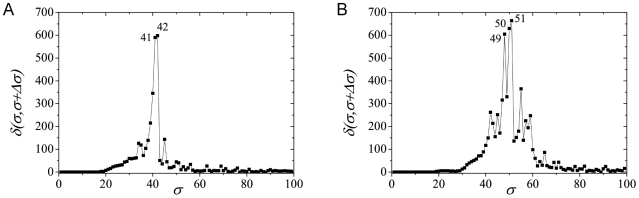
The distance *δ(σ,σ+Δσ)* between networks for successive similarities at the maximal value, with *Δσ* = 1, in the case of: a) Acetyl at *σ = σ_max_* = 42%; b) UDP at *σ = σ_ma_* = 51%.

The presented interpretation of the influence of *σ* on *δ*(*σ,σ+*Δ*σ*) is corroborated by other network measures. Let us consider how *N_c_*, the size of the largest connected component in the network, depends on *σ*. This is illustrated in Figures 2a and 2b for the Acetyl and UDP networks, respectively (see also [35]). In both figures we notice a rapid decrease of *N_c_* in a relatively narrow interval of values of *σ*. This effect is related to the detachment of large groups of nodes from the main cluster as the restriction on establishing links between nodes is increased. As anticipated in the previous section, the curves follow the same qualitative features as those for the Erdös-Rényi networks as a function of the attachment probability *p* close to *p_c_*. Figures S1 and S2 illustrates how *δ* and *N_c_* depend on *p* for networks with the average size of the analyzed PSN's (*N = 256*) and also in the limit of large *N* (see also Text S1).

Hereafter, we will consider the dendrograms, the neighborhood matrices, and the usual representation of the network associated with the proteins listed in Table 1 for the values of *σ* such that the distance shown in Figures 1a and 1b assumes a maximum value. Concerning UDP, the figures are not shown, since they were already presented in a previous paper [35], in which the criterion for setting up the range of *σ* that reveals the modular structure of network was based on the region of transition associated with *C* and 〈*d*〉. It is important to call the attention to the fact that the criterion based on the distance *δ*(*σ,σ+*Δ*σ*) reveals in a much more precise way, in comparison to *C* and 〈*d*〉, the value of *σ* in which the modular structure is observed.

**Figure 3:**
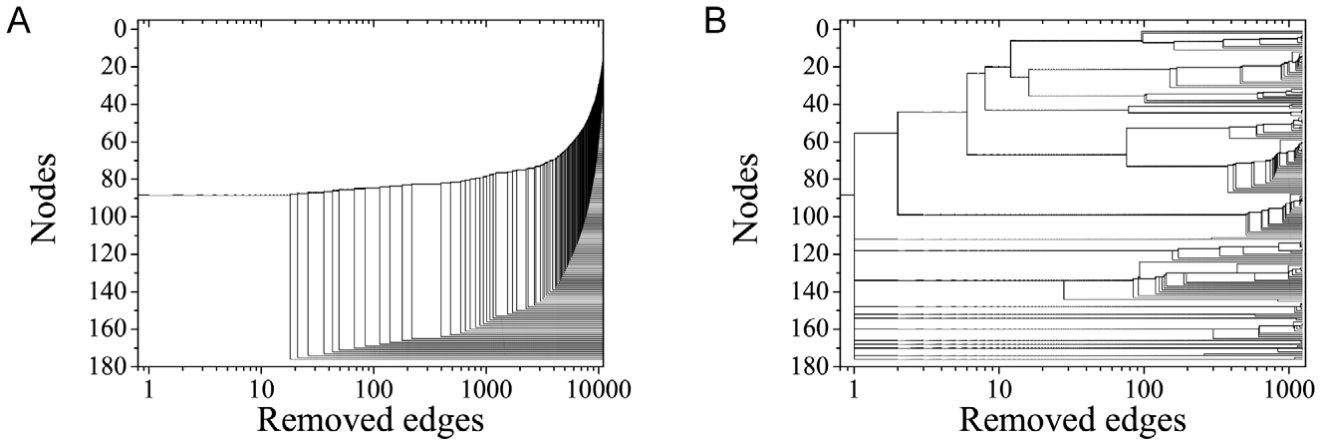
The dendrogram produced by the successive elimination of links with largest value of betweeness in the case of Acetyl: a) for *σ* = 30%<42%; b) for *σ = σ_max_* = 42% that reveals the modular structure of the network.

The influence of *σ* on the network structure can be better appreciated by comparing two dendrograms in Figure 3 for the Acetyl networks at *σ* = 30% and *σ = σ_max_* = 42%. In the first situation (Figure 3a), the very large number of edges does not allow one to perceive the system modular structure. Accordingly, the NGA based on *b_ij_* is characterized by a progressive detachment of small groups of nodes from the original giant cluster. In turn, the dendrogram for *σ = σ_max_* (Figure 3b) reveals a lot of structure. It starts, at *r* = 0, with some already isolated clusters, corresponding to the modules that were detached at *σ = σ_max_*, *σ* = 45%, and *σ* = 48%. Then, we note the separation of a large cluster at a low value of *r*, which is caused by the elimination of the few bonds with very large betweenness degree connecting nodes of the different modules. Such cluster detachment is exactly the same one produced by increasing the value of *σ* to 42%, causing the absolute *δ*(*σ,σ+*Δ*σ*) maximum in Figure 2a. The subsequent elimination of bonds leads to further branching in the dendrogram, some of which can be related to local maxima in the *σ>σ_max_* region of the *δ*(*σ,σ+*Δ*σ*)×*σ* plot.

Dendrograms evaluated at intermediate *σ* values, e.g., *σ* = 40%, are able to clearly identify network modules corresponding to those clusters detached from the giant cluster by selecting *σ* close to this peak value at *σ_max_*. However, the picture that emerges for those clusters that detach at larger values is still rather blurred.

**Figure 4:**
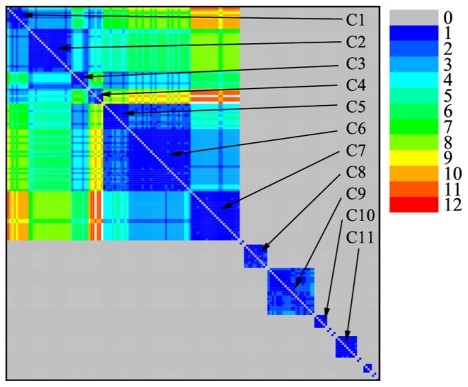
The neighborhood matrix with the 11 modules for Acetyl at *σ = σ_max_* = 42%.

As anticipated in the previous section, let us put together supplementary results in the dendrogram construction to display the network modular structure with the help of the neighborhood matrix 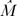. To avoid line crossings in the dendrogram, the order at which the isolated nodes are drawn for the largest value of *r* does not necessarily follow the original numbering. This ordering defines a new node labeling which leaves untouched the network topology. If we now use a color code to represent 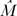 with relabeled nodes, the modularity structure becomes quite clear, as shown in Figure 4. Running from blue (immediate neighbors) to red (farthest apart nodes), the colors clearly indicate how the nodes are grouped into modules, as well as the existence of sub-clusters within the modules and the average distance between nodes in distinct modules. Note that we use gray to indicate the value *d(i,j)* = 0, so that the communities that have been detached from the main cluster at lower values of *σ* appear isolated from one another in the color diagram. We identify 11 modules (C1–C11), the biological significance of which will be discussed below. We note also a number of isolated nodes or small sub-graphs that do not constitute a module on its own. Figure 4 shows the color plot for the neighborhood structure for the Acetyl network at *σ = σ_max_*. It is relatively easy to infer the structure of the dendrograms from the position of the modules. It is important to stress that both graphs not only show the modular structure of the network, but also clearly depict how the retrieved communities are related to each other.

**Figure 5:**
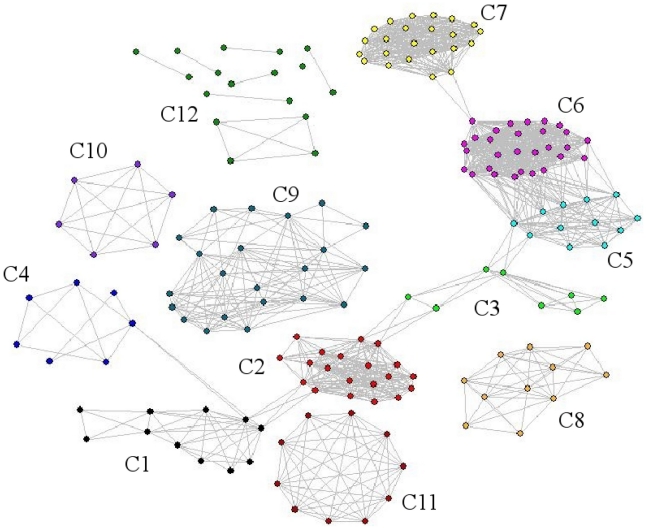
The standard network representation of Acetyl at *σ = σ_max_* = 42% (using Pajek package) with the communities that were indicated in **Figure 4**. We label as C12 the small sub-graphs and isolated nodes that do not constitute a biologically meaningful community.

The information obtained from the described procedure can be also used for the usual network representation formed by nodes and links. In Figure 5, we draw such representation for the Acetyl network at *σ = σ_max_*. Here, the colors used to draw the nodes represent the different communities they belong to. The set of isolated nodes and small sub-graphs is characterized by the C12 label.

This discussion shows that the proposed method allows us to find the most relevant networks, namely those at critical values of *σ_cr_*. These values, where the network topology changes abruptly, correspond to optimal choices between inter-community edge elimination (noise effect) and intra-modules bond preservation (valuable information). They allow us to identify distinct communities, which can be related, then, to the sets of organisms to which the proteins belong (see also Figures S3, S4, S5, S6 and S7). We observe that *σ_max_* corresponds to the particular *σ_cr_*, where *δ*(*σ_cr_,σ_cr_+*Δ*σ*) reaches the largest value.

We show in Table 2 the values of *σ_max_*, the number of nodes, and the number of communities obtained for each of the five enzyme networks. In the case of UDP, we observe the highest *σ_max_* value, indicating that, in the case of this protein, the disassembling of the original, completely connected cluster happen at higher values of similarity. This is a protein with a central role in the chitin synthesis, and, consequently, it is not surprising that it shows the greatest degree of sequence conservation throughout evolution, among the proteins studied in this work. This suggests additional features of the method discussed here, in that there is a relationship between the *σ_max_* value, the degree of sequence conservation of proteins (a structural feature), and their centrality in metabolic networks (a functional feature).

**Table 2:** Summary of the results for each of the five enzyme networks: values of *σ_max_* corresponding to the largest peaks in the graphs *δ×σ*; number of nodes; number of distinct organisms; and the number of distinct communities.

Protein	*σ_max_*	# nodes	# organisms	# communities
Acetylglucosamine phosphate deacetylase	42	176	88	12
Glucosaminephosphate isomerase	40	313	209	5
Hexosaminidase	37	238	67	10
Phosphoglucoisomerase	37	501	332	6
UDP-acetylglucosamine pyrophosphorylase	51	327	245	7

### Biological interpretation

It is relevant to notice that, up to this point, all discussed results have been obtained without any previous knowledge of phylogenetic classification. We only constructed computer routines to proceed with the data analysis, network construction, and network analysis, leading to community identification.

If we now interconnect the results discussed above with taxonomic and phylogenetic data, sound biological information can be promptly retrieved by these computational routines, without using biological assumptions other than those incorporated by BLAST in the production of its outputs.

The Acetyl modules that can be identified at *σ = σ_max_* (Figure 4) correspond, in a clear and rather precise manner, to bacterial phyla and/or classes (and even orders, in some communities). As already discussed, we restricted our analysis to those phyla due to the fact that most of the protein sequences in the database were derived from this biological domain. All cyanobacterial representatives formed only one and exclusive group retrieved in the module C8(a). Furthermore, there are six communities [C3(a), C4(a), C5(a), C6(a), C7(a), C10(a), C11(a)] that are formed exclusively by representatives of one single bacterial phyla or class and, in some cases, order: community C3(a) is exclusively formed by species of the same bacterial order (Mollicutes); community C4(a) are all composed of representatives of Actinobacteria, high G+C Gram-positive monoderm bacteria, of the same class (Actinomycetales); community C5(a) exclusively includes alpha-proteobacteria of the class Rhodobacterales; and community C11(a) contains only species of Firmicutes, low G+C Gram-positive monoderm bacteria, belonging to the very closely related orders Bacillales and Lactobacillales. Although not entirely composed of representatives of the same phyla, 18 out of 20 nodes (90%) of community C2(a) are from the same bacterial phyla (Proteobacteria) and 16 (80%) are from the most phylogenetically related classes of beta- and gamma-proteobacteria [47].

Four modules are retrieved in the Glucosaminephosphate isomerase (gluco) network at *σ = σ_max_* = 40%, and, as in the case of UDP and Acetyl, most of them correspond to single bacterial phyla and/or classes (and even orders): community C2(g) is exclusively composed by bacterial representatives of phyla Firmicutes of only two classes: Bacillales and Lactobacillales; community C4(g) is entirely formed by sequences of the order Alteromonadales of the class gamma-proteobacteria; and 21 out of 23 sequences (91.3%) of community C3(g) are representatives of the phyla Proteobacteria (Figures S5a, S6a, and S7a).

A total of 9 modules occur in the Hexosaminidase (hexo) network at *σ = σ_max_* = 37% and three of them, which contain the greatest number of nodes, are almost exclusively formed by only one bacterial phyla or class: Community C1(h) is composed of 97 nodes, of which 95 (98%) are representatives of phyla Proteobacteria; community C2 is almost exclusively formed by species of the class alpha-proteobacteria; and community C4(h) contains only members of the most phylogenetically related classes of beta- and gamma-proteobacteria [47]. The other communities are all composed by few nodes corresponding to species of distinct phyla (Figures S5b, S6b, and S7b).

Five modules occur in the Phosphoglucoisomerase (phospho) network at *σ = σ_max_* = 37% and, similarly to the other enzymes of the chitin metabolic pathway, there is a rather strict correspondence between these modules and bacterial phyla. Community C1(p) is mainly composed by cyanobacterial representatives (71%), community C2(p) is almost exclusively formed by species of Firmicutes (96.4%), and the very large community C5(p), with 328 nodes, is mainly represented by sequences of Proteobacteria (76%) (Figures S5c, S6c, and S7c).

Finally, UDP can be decomposed into 6 clearly identified modules C1(u)–C6(u), as has been shown previously [35]. C1(u) is composed by 16 nodes, 14 (87.5%) of which are protein sequences from representatives of the phylum Cyanobacteria. One of the nodes corresponds to a sequence from a species of Deinococcus-Thermus, a Gram-negative diderm bacterial group of extremophiles that is closely related to Cyanobacteria [48]. C2(u) contains 135 nodes and, among them, 132 (97.8%) are sequences from species of both beta- and gamma-proteobacteria, which are considered to be more closely related to each other than to any other proteobacterial class [47]. C3(u) is entirely constituted by 80 sequences from Firmicutes species, of three phylogenetically related orders: Bacillales, Lactobacillales, and Clostridiales. C4(u) contains 33 vertices, of which 31 (93.4%) are sequences from the presumed monophyletic group of alpha-proteobacteria [47]. C5(u) is entirely formed by sequences from Actinobacteria, all from the same order: Actinomycetales. Finally, C6(u) comprises only nine nodes from the putative monophyletic group of epsilon-proteobacteria [47], all from the same order: Campylobacterales.

Usually, all the main bacterial phyla (Actinobacteria, Cyanobacteria, Firmicutes, Proteobacteria) and, in some cases, also some bacterial classes (alpha-, beta- and gamma-Proteobacteria), corresponded totally (100%), or with a substantial number of representatives (>70%), to the modules formed as a result of the complex network analysis of the proteins of the chitin metabolic pathway. Even when there were few completely sequenced genomes exhibiting one of the studied proteins, all the representatives of the same phyla were generally grouped together in the same community.

In each of the protein networks, the nodes with the highest degree numbers, or hubs, occurred inside the same community. Although these hubs were not the same in the five different protein networks, many of them were from the same bacterial species for distinct proteins, e.g. *Yersinia pestis* for gluco, hexo, and UDP; *Escherichia coli* for acetyl, hexo, and UDP. In contrast to all other proteins, the hubs in the gluco network were mainly archeal representatives.

### Internal consistency and comparison with phylogenetic methods

The results for a phylogenetic analysis provided by several distinct methods do not necessarily agree with each other, as one can verify by a direct comparison of the outputs produced by each of them. Although we will not make here a detailed comparison between our method and other procedures used to recover phylogenetically useful information, but limit ourselves to take into account the classification obtained for the original dataset, we are in a position to discuss the internal consistency of our method.

The modules defined by the five different enzymes do not necessarily agree with each other for two distinct reasons: first, because not all organisms possess all the enzymes involved in the chitin pathway. This is already clear by the different number of nodes in each of the five networks. Second, because during the course of evolution some enzymes may have suffered more changes than the corresponding enzyme in other organisms, so that the similarity index *S_ij_* between organisms *i* and *j* may take distinct values for two different enzymes. Such quantitative changes may alter the way the organisms are arranged into communities in the corresponding networks. In particular, it may happen that different networks produce distinct number of communities because different enzymes may have changed to a different extent in the organisms, so that one organism may belong to different communities in the networks obtained for different enzymes. Since the same protein may have been independently inserted more than once into the database during the process of uploading the recordings available in Genbank, we have found that the number of distinct organisms in each of the 5 networks is always smaller than the number of nodes (Table 2). We avoided, then, to advance biological hypotheses before the elimination of the isoforms.

The congruence of the classification provided by the distinct networks obtained for the five enzymes of the chitin metabolic pathway was evaluated by means of the congruence index *G*(*ϕ*,*ψ*), defined in the previous section as the ratio *Q*(*ϕ*,*ψ*)/*R*(*ϕ*,*ψ*). For instance, if we take into account the classifications provided by acetyl and UDP we notice that they consist, respectively, of 176 and 327 nodes, which actually correspond to 88 and 245 organisms, distributed into 12 and 7 communities (Table 2). The number of common organisms and correct matches are *R*(*ϕ*,*ψ*) = *44* and *Q*(*ϕ*,*ψ*) = *40*, so that *G*(*ϕ*,*ψ*) = 0.91. The results for the other pairs of networks are shown in Table 3.

**Table 3:** Values of congruence obtained after pair-wise comparison of the phylogenetic analysis provided by two different networks.

	A	G	H	P	U
A		0.79	0.73	0.93	0.91
G	0.79		0.69	0.83	0.87
H	0.73	0.69		0.90	0.79
P	0.93	0.83	0.90		0.95
U	0.91	0.87	0.79	0.95	

The average value of the entries in the table is 84%. Abbreviations: A, acetyl; G, gluco; H, hexo; P, phosphor; U, UDP.

**Figure 6:**
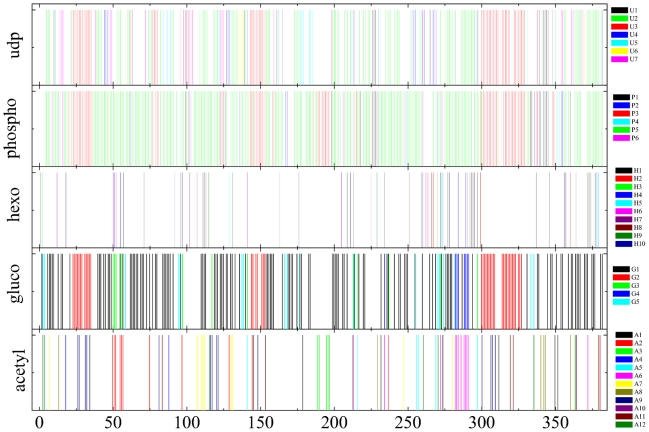
Series of spikes representing the 382 organisms present in each one of the 5 selected enzymes associated with the chitin metabolic route. Along each series of spikes, color identifies the group the organisms belong to. There is no color correspondence between two network classifications.

In Figure 6 we display the results obtained from the community identification for all 5 networks (In Figure S8, one can see the same figure with the horizontal axis expanded for better visualization). In this representation we take into account only the number of 382 distinct organisms represented by the original 1695 entries. The used association (number, organism) is available in the Supplementary Information. Each of the five networks is represented by a horizontal sequence of spikes, which identify which organisms are present in each network. Within a given network, the color of the spikes identifies to which community the organism belongs. Since different networks have different numbers of communities, there is no color correspondence between distinct network classifications. Congruence can be measured by the same color criterion: if the spikes corresponding to organisms *i* and *j* have the same color in network *ϕ* and network *ψ*, the classification provided by *ϕ* and *ψ* is congruent, even if the common color in *ϕ* is different from the common color in *ψ*.

The subsequent steps of our research program comprise a detailed comparison between the results obtained with the complex network approach reported in this paper and the outcomes of other methods used to analyze phylogenetic relationships based on molecular data. Although this is a computationally complex task [49], [50], the results of which need to be discussed in another work, it is possible to advance that preliminary results for a much smaller data set than that used herein are promising – namely, data about chitin synthase, another protein of the chitin metabolic pathway. Using the PAUP 4.0 program [51] to perform distance, likelihood, and parsimony analyses, and Mr. Bayes 3.02 [52] to perform Bayesian analysis, we provided a comparison between the proposed phylogenetic classification with those based on the Bayesian, distance, likelihood, and parsimony criteria. The results shown in Table 4 are based on the same congruence criterion we used to compare the data in Table 3. In particular, the average congruence of our method with the four other methods reaches 69%, while the average taken over the six pair-wise comparisons among the four methods (B, D, L. P) reaches only 60%. These results allow us to conclude that the methodology reported in this paper is as reliable as those commonly used methods.

**Table 4:** Values of congruence obtained after pair-wise comparison of the phylogenetic analysis based on chitin synthase sequences provided by five different methods: Bayesian (B), distance (D), likelihood (L), parsimony (P), and the network method introduced herein (N).

	B	D	L	P	N
B		0.74	0.82	0.51	0.82
D	0.74		0.69	0.54	0.54
L	0.82	0.69		0.59	0.82
P	0.51	0.54	0.59		0.59
N	0.82	0.54	0.82	0.59	

Average congruence of N with the four other methods = 69%. Average taken over the six pair-wise comparisons among the four methods (B, D, L, P) = 60%.

### Conclusions

This work reports a method based on complex network theory that can recover information about the evolutionary relationships between organisms, as expressed in the similarities and differences between their protein sequences, which is useful for phylogenetic inference. The system interaction network constructed is based on protein similarity as the meaningful criterion to place an edge between two nodes. Each node in the network is a specific protein sequence and the placement of edges depends on a threshold value *σ*, related to the protein similarity required to such a placement.

We performed a comparative study of the enzymes related to the chitin metabolic pathway in completely sequenced genomes of extant organisms of the three life domains, Archaea, Bacteria, and Eukarya, in order to show how the information derived from the network structure and statistics can uncover phylogenetic patterns. The results concerning phylogenetic classification discussed in this paper are mainly based on the modular character of protein similarity networks. Once the critical value of *σ* (*σ_cr_*) using the distance measure *δ(α,β)* is found, we can choose the optimal network for community detection, namely, that in which the network topology changes abruptly, corresponding to optimal choices between inter-community edge elimination (noise effect) and intra-modules bond preservation (valuable information). Although the NGA can be used to identify communities for any value of *σ*, it is in this optimal network that the best results can be achieved with regard to the identification of distinct communities, which can be related, in turn, to the sets of organisms to which the proteins belong.

With this method, sound biological information can be promptly retrieved by computational routines, without using biological assumptions other than those incorporated by BLAST. Usually, all the main bacterial phyla and, in some cases, also some bacterial classes corresponded to a great extent (70%–100%) to the modules obtained by means of the complex network analysis of the proteins of the chitin metabolic pathway. Therefore, the method reported here can be used as a powerful tool to reveal relationship patterns among both organisms we have knowledge about and organisms about which we do not have much information available.

We provided results showing the internal consistency of the results obtained through our method for the data corresponding to five different enzymes. Despite the different rates of changes suffered by these enzymes during evolution, we found 84% of matches for community pertinence when comparisons between the results were performed. Moreover, a preliminary comparison between the results obtained with the complex network approach reported here and the outcomes of methods based on Bayesian, distance, likelihood, and parsimony criteria suggests that the methodology reported in this paper is as reliable as these commonly used methods.

There are, however, some possible advantages of the complex network method when compared to these other methods. One of them concerns the fact that we can determine the value of *σ* in which the complex network retrieve most of the phylogenetic information available in the data set. Second, even though all these methods use substitution matrices – including ours –, the complex network method is not dependent upon patterns inferred from the detailed study of any organisms.

The next steps in our research program will be the application of the method presented here to new sets of protein sequences, a more thorough comparison of the results obtained through our complex network approach with the outcome of other methods employed to retrieve information from molecular data that is useful for phylogenetic inference, and the application of our method to address relevant research questions within different fields of biology.

## Supporting Information

Figure S1 Graphs of δ(p,p+Δp) as function of p for N-nodes ER networks (G(N,p)), where p indicates the probability of introducing an edge between any pair of nodes. For the sake of a better comparison, p is restricted to the interval [0,5pc = 5/N] for any value of N. The solid line indicates the average behavior (10 samples when N = 256 (a), and 3 samples when N = 4096 (b)), while dashed lines illustrate the typical behavior of a single sample. The values of p where peaks are present are much smaller than the corresponding values of σ in PSN. When N = 256, the typical order of magnitude of the protein networks, distinct modules of comparatively large size are individually formed. The several peaks indicate the values of p at which different modules merges, producing a similar landscape to that observed in the PSN networks. The maximum of the averaged curve occurs at values of p>pc. When N increases (b), the fluctuations in the values of δ(p,p+Δp) decrease and the maximum is displaced to the left, becoming closer and closer to pc. The peak is much sharper, and the slope of the curve in its neighborhood is much larger. This indicates that the number of components of relatively large size is reduced, and that all smaller clusters start to merge with the largest component in very narrow interval of values of p.
(0.11 MB TIF)

Figure S2 Behavior of the size of the largest connected component Nc as function of p for ER networks G(N,p). As in Fig. S7, for any value of N, p is restricted to the interval [0,5pc = 5/N], while solid and dashed lines indicate average and single sample behavior. For both values of N, the values of Nc at pc are close to the expected value (Nc(pc)≈N2/3). However, the slope of the curve is much larger when N = 4096, what can be related to the exponential increase in Nc(p>pc) in the limit N→∞ and the sharpness of the peak of δ(p,p+Δp).
(0.08 MB TIF)

Figure S3 The size of the largest cluster (N_c_) versus the threshold similarity σ: a) Gluco; b) Hexo; c) Phospho.
(0.16 MB TIF)

Figure S4 The distance δ(σ,σ+Δ σ) between networks for successive similarities at the maximal value in the case of: a) Gluco at σ = σ_max_ = 40%; b) Hexo at σ = σ_max_ = 37%; c) Phospho at σ = σ_max_ = 37%.
(0.25 MB TIF)

Figure S5 The dendrogram associated with the elimination of links with largest value of betweeness in the case of: a) Gluco at σ = σ_max_ = 40%; b) Hexo at σ = σ_max_ = 37%; c) Phospho at σ = σ_max_ = 37%.
(0.38 MB TIF)

Figure S6 The neighborhood matrix with the communities for: a) Gluco at σ = σ_max_ = 40%; b) Hexo at σ = σ_max_ = 37%; c) Phospho at σ = σ_max_ = 37%. The presence of other high peaks for the Gluco network shown in Fig.S2a indicates that the complete separation of communities C1 and C2, and C3 and C4 is achieved only at σ = 50%.
(2.18 MB TIF)

Figure S7 The standard representation of each enzyme network (using the Pajek package) displaying the communities that were indicated in Fig. 4a, 4b and 4c respectively: a) Gluco at σ = σ_max_ = 40%; b) Hexo at σ = σ_max_ = 37%; c) Phospho at σ = σ_max_ = 37%. One extra label has been added in each panel to denote the set of isolated nodes and small sub-graphs. Note that figures were drawn for the value σ_max_ and module separation occurs only at σ_max+1_, so that these set is about to be separated from the main cluster.
(3.61 MB TIF)

Figure S8 Same as in Fig. 6 of the published material, but the horizontal axis has been expanded for the sake of a better visualization. Color codes and network order is the same as in the published material.
(1.76 MB TIF)

Text S1 Supplementary material for the paper “Detecting Network Communities: An Application to Phylogenetic Analysis.”
(0.03 MB DOC)
