# Supplementary figures and images for: Detecting Network Communities: An Application to Phylogenetic Analysis
Source: PLoS Comput Biol. 2011 May 5;7(5):e1001131. doi: 10.1371/journal.pcbi.1001131 (PMC3088654; doi:10.1371/journal.pcbi.1001131)

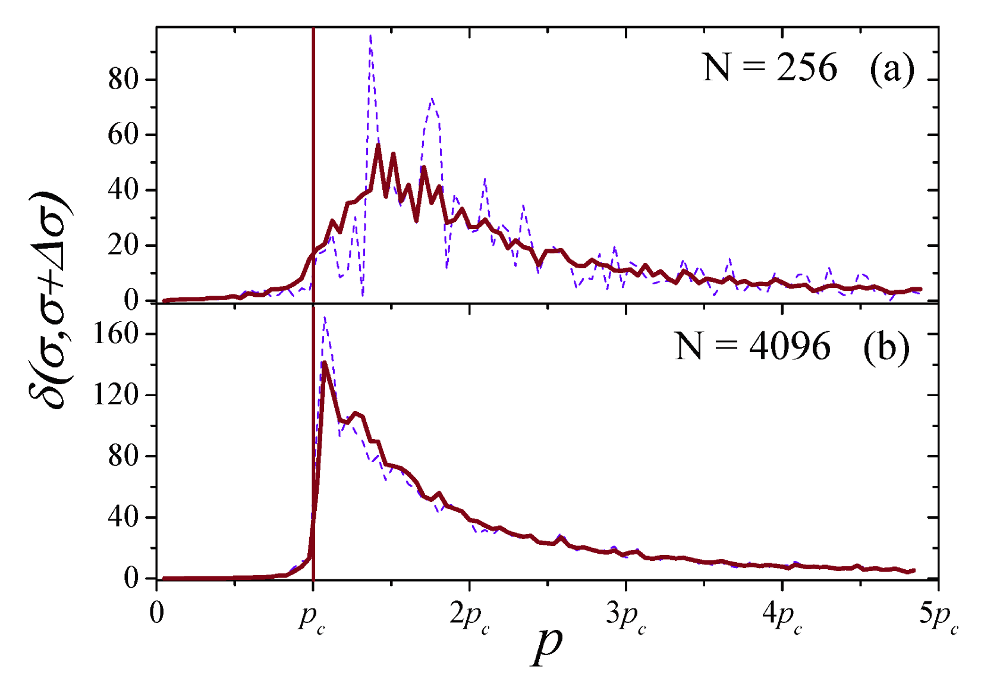

Supplement: Figure S1 — Graphs of δ(p,p+Δp) as function of p for N-nodes ER networks (G(N,p)), where p indicates the probability of introducing an edge between any pair of nodes. For the sake of a better comparison, p is restricted to the interval [0,5pc = 5/N] for any value of N. The solid line indicates the average behavior (10 samples when N = 256 (a), and 3 samples when N = 4096 (b)), while dashed lines illustrate the typical behavior of a single sample. The values of p where peaks are present are much smaller than the corresponding values of σ in PSN. When N = 256, the typical order of magnitude of the protein networks, distinct modules of comparatively large size are individually formed. The several peaks indicate the values of p at which different modules merges, producing a similar landscape to that observed in the PSN networks. The maximum of the averaged curve occurs at values of p>pc. When N increases (b), the fluctuations in the values of δ(p,p+Δp) decrease and the maximum is displaced to the left, becoming closer and closer to pc. The peak is much sharper, and the slope of the curve in its neighborhood is much larger. This indicates that the number of components of relatively large size is reduced, and that all smaller clusters start to merge with the largest component in very narrow interval of values of p. (0.11 MB TIF) [file pcbi.1001131.s001.tif]

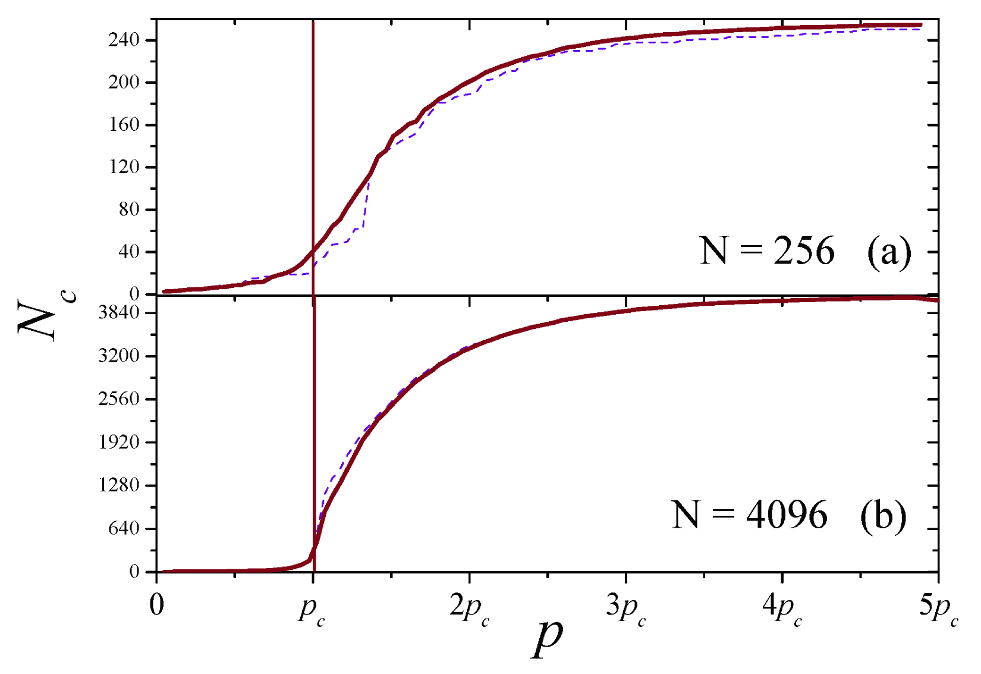

Supplement: Figure S2 — Behavior of the size of the largest connected component Nc as function of p for ER networks G(N,p). As in Fig. S7, for any value of N, p is restricted to the interval [0,5pc = 5/N], while solid and dashed lines indicate average and single sample behavior. For both values of N, the values of Nc at pc are close to the expected value (Nc(pc)≈N2/3). However, the slope of the curve is much larger when N = 4096, what can be related to the exponential increase in Nc(p>pc) in the limit N→∞ and the sharpness of the peak of δ(p,p+Δp). (0.08 MB TIF) [file pcbi.1001131.s002.tif]

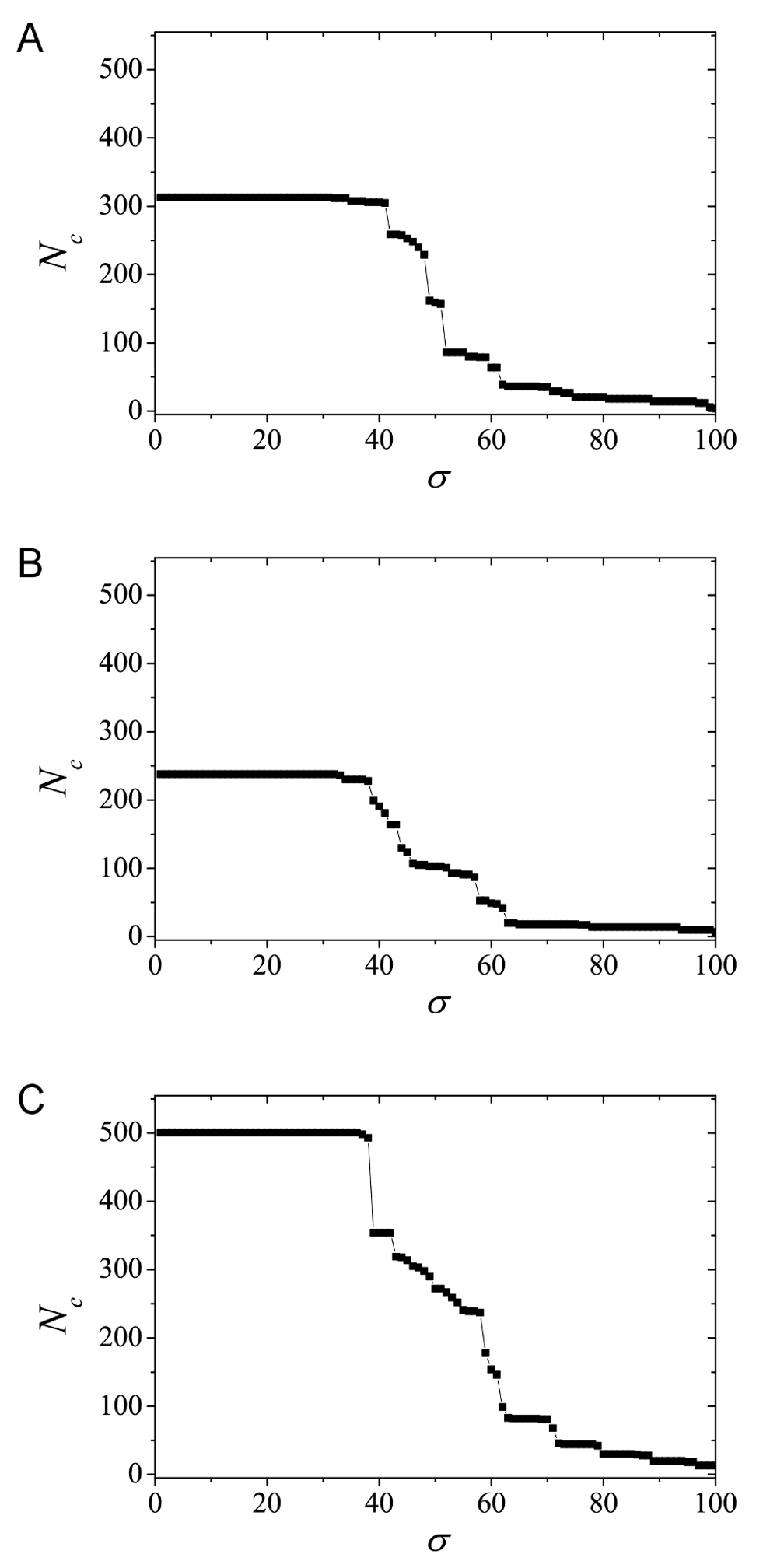

Supplement: Figure S3 — The size of the largest cluster (Nc) versus the threshold similarity σ: a) Gluco; b) Hexo; c) Phospho. (0.16 MB TIF) [file pcbi.1001131.s003.tif]

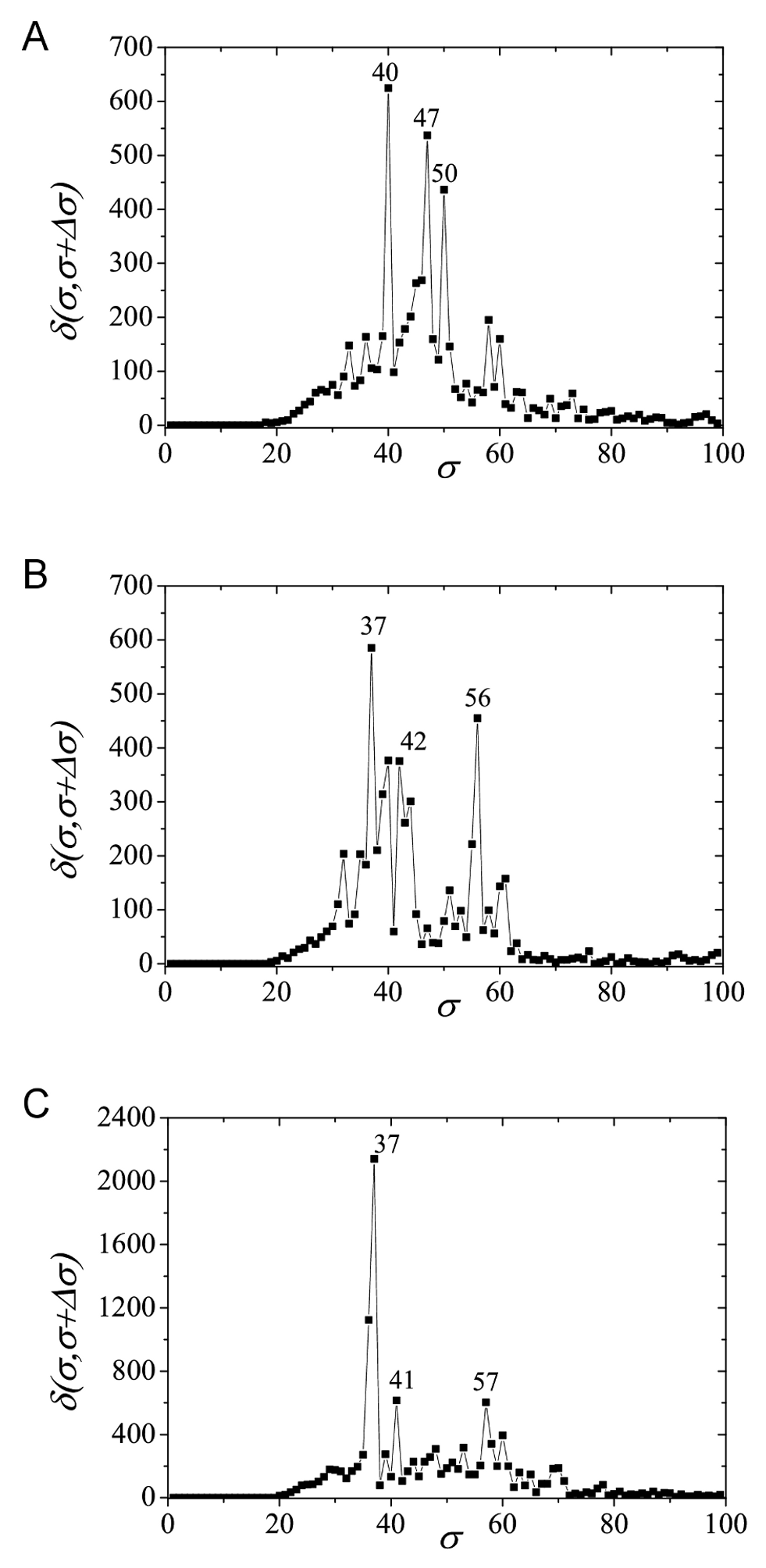

Supplement: Figure S4 — The distance δ(σ,σ+Δ σ) between networks for successive similarities at the maximal value in the case of: a) Gluco at σ = σmax = 40%; b) Hexo at σ = σmax = 37%; c) Phospho at σ = σmax = 37%. (0.25 MB TIF) [file pcbi.1001131.s004.tif]

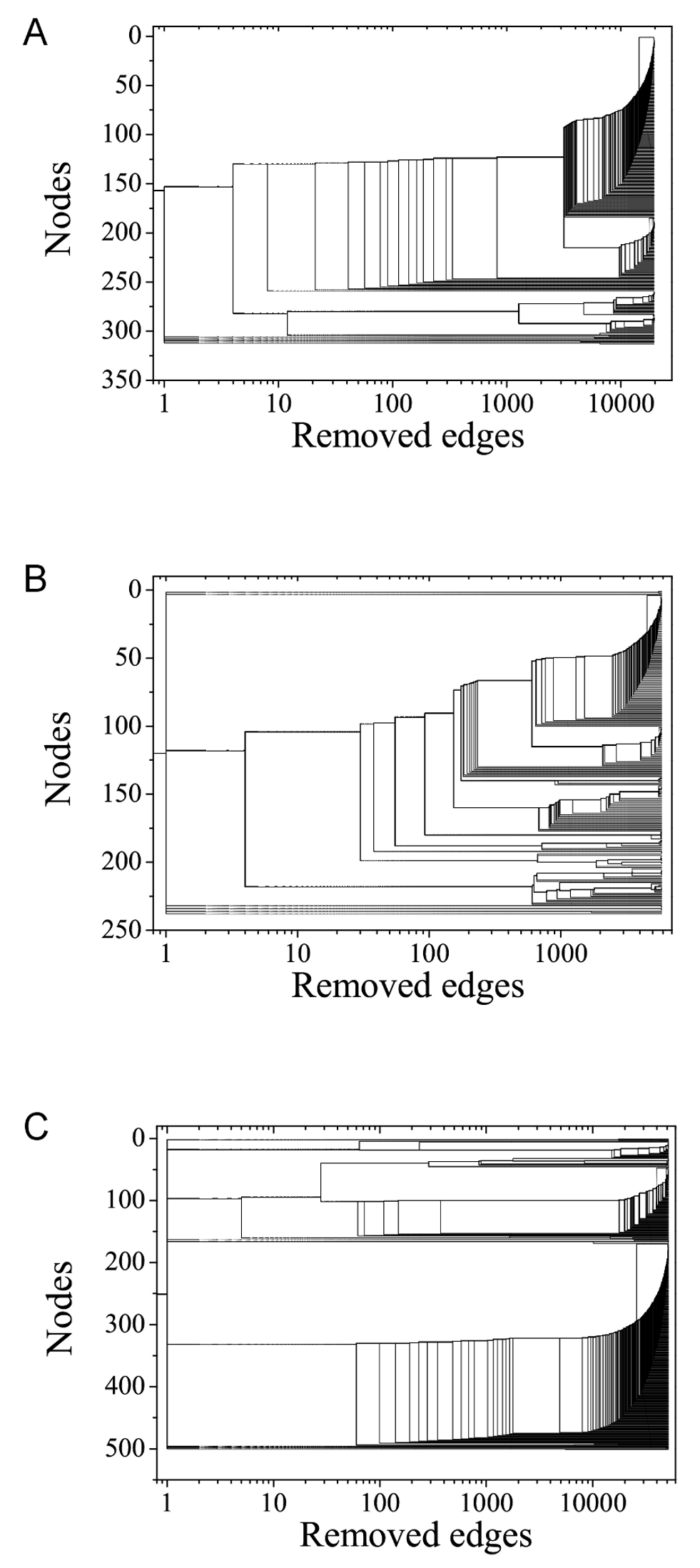

Supplement: Figure S5 — The dendrogram associated with the elimination of links with largest value of betweeness in the case of: a) Gluco at σ = σmax = 40%; b) Hexo at σ = σmax = 37%; c) Phospho at σ = σmax = 37%. (0.38 MB TIF) [file pcbi.1001131.s005.tif]

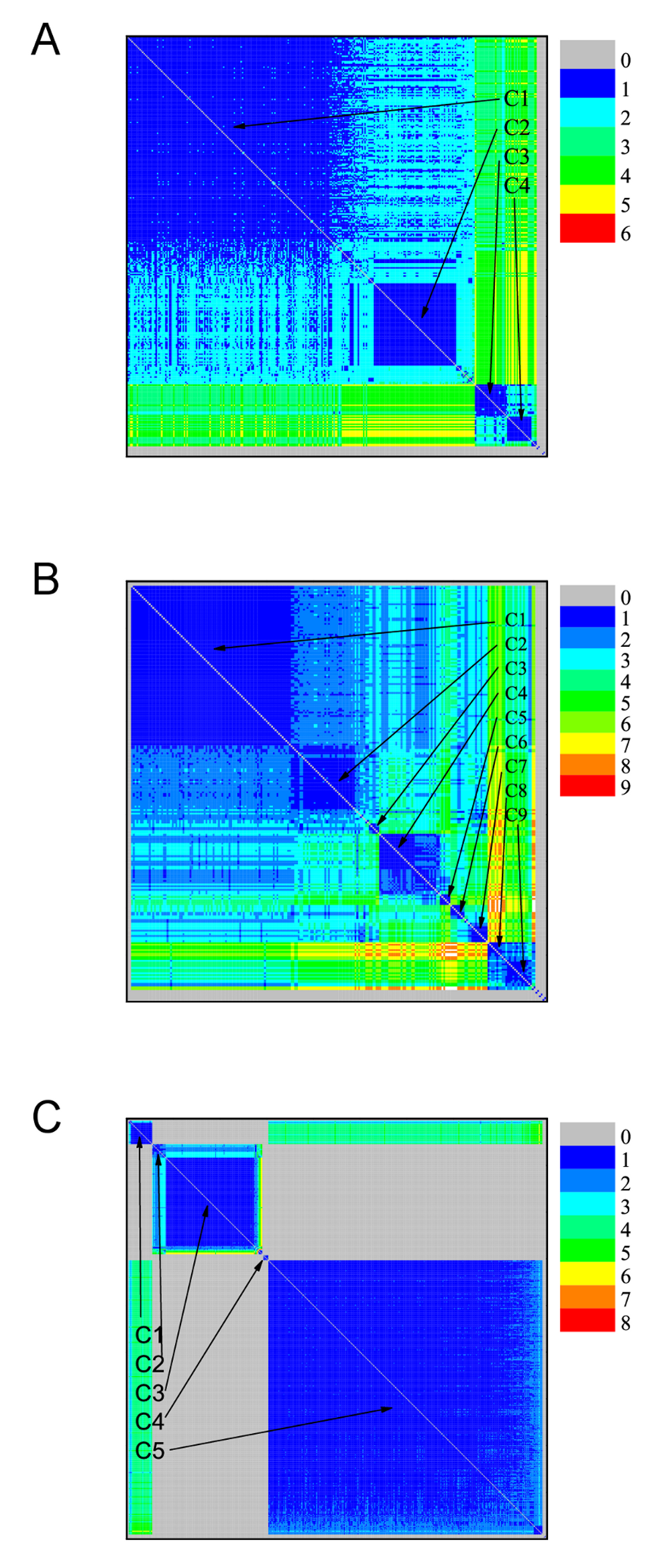

Supplement: Figure S6 — The neighborhood matrix with the communities for: a) Gluco at σ = σmax = 40%; b) Hexo at σ = σmax = 37%; c) Phospho at σ = σmax = 37%. The presence of other high peaks for the Gluco network shown in Fig.S2a indicates that the complete separation of communities C1 and C2, and C3 and C4 is achieved only at σ = 50%. (2.18 MB TIF) [file pcbi.1001131.s006.tif]

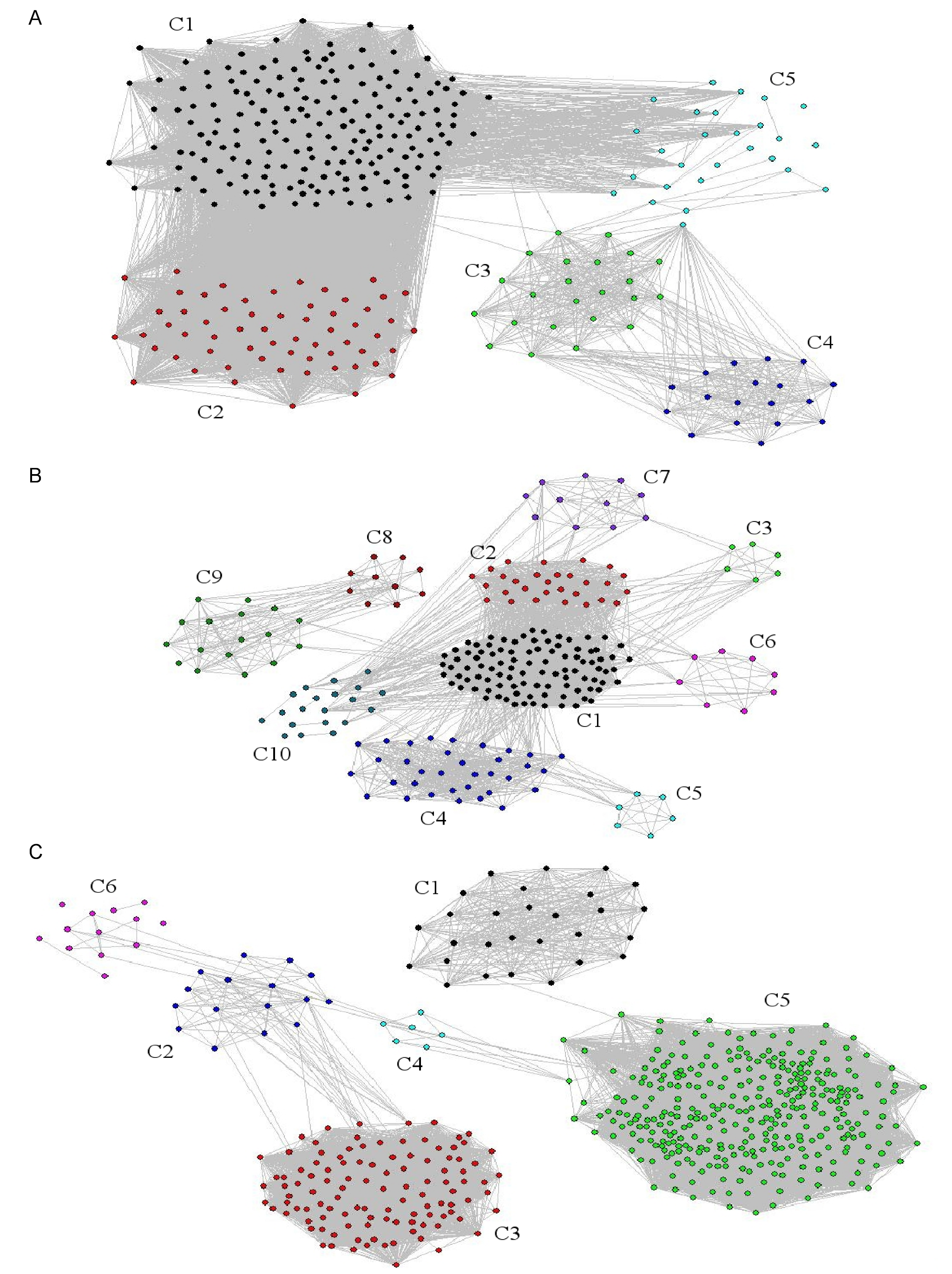

Supplement: Figure S7 — The standard representation of each enzyme network (using the Pajek package) displaying the communities that were indicated in Fig. 4a, 4b and 4c respectively: a) Gluco at σ = σmax = 40%; b) Hexo at σ = σmax = 37%; c) Phospho at σ = σmax = 37%. One extra label has been added in each panel to denote the set of isolated nodes and small sub-graphs. Note that figures were drawn for the value σmax and module separation occurs only at σmax+1, so that these set is about to be separated from the main cluster. (3.61 MB TIF) [file pcbi.1001131.s007.tif]

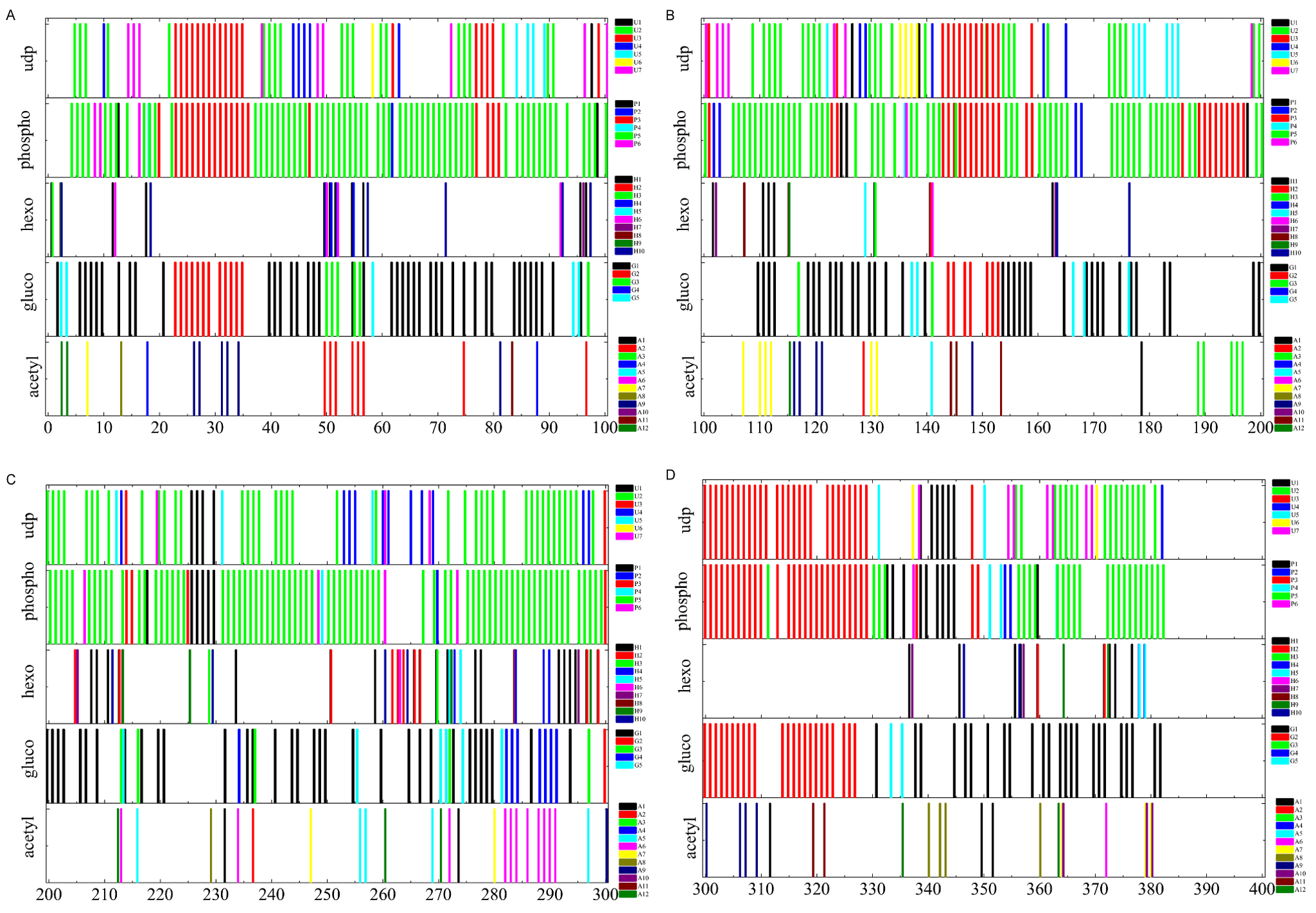

Supplement: Figure S8 — Same as in Fig. 6 of the published material, but the horizontal axis has been expanded for the sake of a better visualization. Color codes and network order is the same as in the published material. (1.76 MB TIF) [file pcbi.1001131.s008.tif]
