# Supplementary material for: Detecting Network Communities: An Application to Phylogenetic Analysis
Source: PLoS Comput Biol. 2011 May 5;7(5):e1001131. doi: 10.1371/journal.pcbi.1001131 (PMC3088654; doi:10.1371/journal.pcbi.1001131)
Supplement: Text S1 — Supplementary material for the paper “Detecting Network Communities: An Application to Phylogenetic Analysis.” (0.03 MB DOC) [file pcbi.1001131.s009.doc]

Supplementary material for the paper “Detecting Network Communities: An Application to Phylogenetic Analysis”

In the supplementary material, we include a series of figures that show the results for the other three enzymes in the data set: Glucosaminephosphate isomerase (Gluco), Hexosaminidase (Hexo), Phosphoglucoisomerase (Phospho). They correspond to the same kinds of result discussed in the manuscript for acetylglucosamine phosphate deacetylase (Acetyl). We also insert figures that illustrate how our method, based on the selection of an optimal value of a threshold measure in a weighted network, is able to identify the formation of a giant component in the neighborhood of the percolation threshold in the case of the random Erdös-Renyi network.
